# Characterization of nonrapid virologic response patients infected with HCV genotype 1 who may relapse after standard therapy with peginterferon plus ribavirin

**DOI:** 10.1111/j.1365-2893.2010.01422.x

**Published:** 2012-02

**Authors:** N Reau, F M Hamzeh, E Lentz, X Zhou, D Jensen

**Affiliations:** 1 University of Chicago Medical CenterChicago, IL; 2 GenentechSan Francisco, CA;; 3 RTI Health SolutionsResearch Triangle Park, NC, USA

**Keywords:** genotype 1, hepatitis C virus, peginterferon, predictive model, relapse, ribavirin

## Abstract

Approximately 50% of patients with hepatitis C virus (HCV) genotype 1 treated with peginterferon alfa-2a/ribavirin discontinue treatment early or experience a suboptimal response despite 48 weeks of therapy. The objective of this analysis was to develop a model to identify nonrapid virologic response (non-RVR) patients who may be candidates for intensified therapy that would increase treatment response. The retrospective analysis included non-RVR patients from four trials of 48-week peginterferon alfa-2a/ribavirin treatment. Patients were grouped into those who cleared virus between weeks 5 and 12 (complete early virologic responders, cEVR) or between weeks 13 and 24 (slow responders). A model was developed to predict relapse at the end of follow-up (week 72). An optimal model was evaluated and compared with current practice by using receiver operating characteristic curves, sensitivity and specificity. In total, 539 non-RVR patients were eligible for analysis of which 72% experienced cEVR and 28% were slow responders. Variables associated with relapse included age, ethnicity, baseline HCV RNA and interval of time to HCV RNA undetectable. The optimal model was most accurate at predicting patients at risk for relapse. The practice of considering treatment intensification (e.g. extending treatment duration) in all slow responders was less accurate but likely most practical. A week 4 HCV <2-log reduction was the earliest but least accurate marker. We developed a model that could identify non-RVR patients at high risk for relapse after 48 weeks of peginterferon alfa-2a plus ribavirin and who may benefit from intensified therapy to reduce this risk of relapse.

Hepatitis C virus (HCV) accounts for approximately 40% of liver-related mortality in the United States [1]. Eradicating the virus may prevent life-threatening complications such as cirrhosis and hepatocellular carcinoma. The current recommended standard of care for chronic hepatitis C (CHC) infection is combination therapy with pegylated interferon alfa plus weight-based ribavirin for 48 weeks. This treatment leads to a sustained virologic response (SVR; defined as absence of detectable HCV RNA by sensitive PCR 6 months after completion of therapy) in 46–52% of patients infected with HCV genotype 1 [2,3]. Thus, approximately 50% of these patients either discontinue treatment early or experience a suboptimal response that can include nonresponse (never achieve undetectable HCV RNA), relapse (recurrence of viraemia despite being HCV RNA undetectable at end of treatment [EOT]) or breakthrough (detectable HCV RNA at EOT after an initial undetectable HCV RNA during treatment).

The ability to predict more accurately the response to treatment is important. In addition to allowing the customization of therapy, predicting response may help reduce the significant adverse events and high cost associated with this therapy. The probability of achieving SVR can be estimated by considering several baseline patient and viral characteristics of which viral genotype (genotype 2 or 3) and pretreatment viral load (<800 000 IU/mL) have emerged from multivariate analyses of large pivotal trials as two major favourable predictors of SVR [2–4].

More recently, early viral kinetics have been shown to be more highly predictive of SVR than baseline factors [5]. Approximately 90% of patients experiencing a rapid virologic response (RVR; undetectable HCV RNA after 4 weeks of treatment) achieve a SVR [5–7]. In those patients who continue to have detectable HCV RNA after week 4, the longer it takes to achieve HCV RNA undetectability the less likely SVR will occur with a 48-week course of combination therapy [8]. Only 21% of patients who experience an early virologic response (EVR; a 2-log decrease in viral load at week 12 from baseline), but do not become HCV RNA undetectable until after week 12, achieve SVR [9]. In contrast, 75% of patients with undetectable HCV RNA by week 12 (complete EVR [cEVR]) achieve SVR [2]. Studies have shown that intensified treatment, including extending therapy for more than 48 weeks, may reduce the rate of relapse in patients infected with HCV genotype 1 who did not achieve RVR [10–12]. Therefore, the objective of this analysis was to develop a model that may be used to identify more accurately the subset of non-RVR patients who may be candidates for intensified treatment and can potentially reduce their chance of relapse after 48 weeks of therapy.

## Materials and methods

### Patients

Analysis included treatment-naϊve adults infected with HCV genotype 1 from four clinical trials [2,3,13,14]. Two of these trials were enriched with patients who had characteristics traditionally associated with a decreased treatment response. The African American trial included 78/106 patients of African American ethnicity [13], and the LATINO trial included 268/567 patients of Latino white ethnicity [14]. All patients enrolled in the trials were anti-HCV antibody positive, had elevated serum alanine aminotransferase (ALT) activity and had quantifiable HCV RNA. The eligible quantity of HCV RNA varied by study; two trials [2,3] used the COBAS® AMPLICOR® HCV Test (Version 2.0; Roche Diagnostics, Branchburg, NJ, USA), one trial [13] used the AMPLICOR® HCV Monitor Test (version 2.0; Roche Diagnostics) and the most recent trial [14] used the High Pure system/COBAS® TaqMan® HCV Monitor Test (Roche Diagnostics). Liver biopsy findings were consistent with the diagnosis of CHC, and only patients with compensated liver disease (Child–Turcotte–Pugh Class A) were included in the analysis. More complete descriptions of the inclusion and exclusion criteria, study design and primary results of the trials have been published elsewhere [2,3,13,14].

Besides the criteria mentioned above, patients were eligible for this analysis if they were randomized to treatment for 48 weeks with peginterferon alfa-2a 180 μg/week plus ribavirin 1000 or 1200 mg/day, initially became HCV RNA undetectable between weeks 5 and 24, were HCV RNA undetectable at week 48 and had a HCV RNA measurement at week 72. All patients who met the above criteria completed 48 weeks of treatment except for two patients who discontinued early because of an adverse event or illness. These two patients were excluded from the analysis.

Depending on the virologic response at week 72, this analysis included non-RVR patients who either achieved SVR or had a confirmed relapse. Patients were then classified as those with cEVR (HCV RNA undetectable at week 12) or those with slow response (HCV RNA undetectable for the first time between weeks 13 and 24).

### Study design

This study involved the *post hoc* analysis of data collected during four randomized, multinational, phase III/IV studies where the primary efficacy end-point was SVR, defined as undetectable serum HCV RNA at the end of a 24-week follow-up phase. Undetectable HCV RNA was defined as <100 copies/mL (Cobas® Amplicor® PCR HCV Test) or <50 IU/mL (Amplicor® HCV Monitor Test) in the three earlier studies [2,3,13], and <28 IU/mL (High Pure System/COBAS® TaqMan HCV Monitor Test) in the most recent study [14].

### Outcome variable and candidate predictors

The outcome variable for this analysis was virologic relapse that was defined as HCV RNA detectable at week 72 following HCV RNA being undetectable at EOT.

Candidate predictors that were potentially associated with relapse and were therefore incorporated into the analysis included: patient demographic and clinical characteristics at baseline, time to first HCV RNA undetectable (i.e. cEVR *vs* 24-week undetectable), HCV RNA reduction at weeks 4, 12 and 24, and average HCV RNA reduction at nadir. Average HCV reduction at nadir (log_10_ per month) was calculated as follows: for cEVR, the average HCV reduction at nadir = (log_10_ HCV_baseline_ − log_10_ HCV_week12_)/3 and for 24-week undetectable, the average HCV reduction at nadir = (log_10_ HCV_baseline_ − log_10_ HCV_week24_)/6. Viral load measurements in copies/mL were converted to IU/mL by using the conversion factor 2.7 (2.7 copies/mL = 1 IU/mL).

### Model development

Logistic regression analysis was used to develop a model to predict relapse at week 72. Descriptive statistics (e.g. percentages and means) of the candidate predictors were reviewed and their relationship with relapse was evaluated using simple logistic regression one at a time. Models were selected starting from a full model which included all candidate predictors and their meaningful interactions (Table 1). A backward selection procedure was used with *P* < 0.20 for elimination. The models obtained after this backward selection were further refined for simplicity and robustness. Percentage dose reduction (as a continuous variable) because of safety reasons was incorporated into the optimal model using a backward selection procedure that eliminated the insignificant (*P* > 0.05) dose reduction variables one at a time.

**Table 1:** Predictors included in the selected models

Full*	Optimal	Week 4
Age (linear and squared terms)	Age (>50 or ≤50 years)	Time to undetectability (cEVR or 24-week undetectable)
Sex (male or female)	Race (African American, Latino white or non-Latino white/other)	Week 4 HCV (log_10_ IU/mL) (per unit increase)
Race (African American, Latino white or non-Latino white/other)	Time to undetectability (cEVR or 24-week undetectable)	
BMI (linear and squared terms)	ALT quotient (per unit increase)	
Weight group (<75 kg or ≥75 kg)	ALT quotient by race interaction	
Baseline ALT quotient (linear term and the interaction with race group)	ALT quotient by time to undetectability	
Cirrhotic classification (cirrhotic or noncirrhotic and the interaction with race group)	Baseline HCV load (log_10_ IU/mL) (per unit increase)	
Time to undetectability (cEVR or 24-week undetectable)	HCV reduction rate: (log_10_ baseline)/(log_10_ week 4) (per unit increase)	
Average HCV reduction at nadir (log_10_ per month, linear and squared terms)	HCV reduction rate: (log_10_ week 4)/(log_10_ week 12) (per unit increase)	
Baseline HCV load (log_10_, linear and squared terms)	Peginterferon alfa-2a dose reduction† (per % increase)	
HCV reduction (log_10_, linear and squared terms)	Ribavirin dose reduction† (per % increase)	
HCV reduction rate (linear and squared terms)		
Indicator for high baseline HCV load (<6.3 log_10_ IU/mL or ≥6.3 log_10_ IU/mL)		
Indicator for high week 4 HCV load (<3.8 log_10_ IU/mL or ≥3.8 log_10_ IU/mL)		
Two-way interactions with time to undetectability		

ALT, alanine aminotransferase; BMI, body mass index; cEVR, complete early virologic responders; HCV, hepatitis C virus.

* The full model is the model that included all the predictors investigated. The more variables included in a model, the higher the predictability of the model. Therefore, the full model offers the maximum predictability.

† Because of safety reasons.

### Model evaluation and comparison

The selected models were evaluated using receiver operating characteristic (ROC) curves, sensitivity (% of patients correctly predicted as relapse) and specificity (% of patients correctly predicted as SVR). To demonstrate further the improvement in prediction, selected models were compared with the practice of intensifying treatment for all slow responders and leaving cEVR patients without treatment beyond 48 weeks.

## Results

### Patient demographics and clinical characteristics

In total, 539 non-RVR patients who became HCV RNA undetectable before week 24 were eligible for inclusion in this analysis, of which 72.0% (*n* = 388) became HCV RNA undetectable during weeks 5 and 12 (cEVR), and 28.0% (*n* = 151) became HCV RNA undetectable during weeks 13 and 24 (slow responders). Demographic and clinical characteristics of the total population are presented in Table 2. The overall SVR rate for the 539 non-RVR patients was 67.2% (362/539). The SVR rates were 77.1% (299/388) for cEVR patients and 41.7% (63/151) for slow responders.

**Table 2:** Demographic and clinical characteristics at baseline for patients in the analysis sample

Characteristic	All patients (*N *=* *539)
Sex, males, *n* (%)	358 (66.4)
Age, years, mean ± SD	45.8 ± 9.6
≤50, *n* (%)	371 (68.8)
>50, *n* (%)	168 (31.2)
Weight, kg, mean ± SD	81.3 ± 17.3
Weight <75 kg, *n* (%)	204 (37.8)
BMI*, kg/m^2^, mean ± SD	27.6 ± 5.2
≤27, *n* (%)	279 (52.4)
27–30, *n* (%)	119 (22.4)
>30, *n* (%)	134 (25.2)
Race/ethnicity	
Non-Latino white, *n* (%)	380 (70.5)
Latino white, *n* (%)	112 (20.8)
African American, *n* (%)	28 (5.2)
Other, *n* (%)	19 (3.5)
ALT quotient, mean ± SD	2.3 ± 1.6
ALT >3 × ULN, *n* (%)	127 (23.6)
HCV RNA, log_10_ IU/mL, mean ± SD	6.3 ± 0.6
≤400 000 IU/mL, *n* (%)	78 (14.5)
>400 000–800 000 IU/mL, *n* (%)	59 (10.9)
>800 000 IU/mL, *n* (%)	402 (74.6)
Cirrhosis classification	
Noncirrhotic, *n* (%)	459 (85.2)

ALT, alanine aminotransferase; BMI, body mass index; HCV, hepatitis C virus; SD, standard deviation; ULN, upper limit of normal.

* Missing data, *n *= 7.

### Model development

Simple logistic regression analysis was performed on each of the candidate predictors as an initial evaluation of a relationship with relapse. Results (Tables 3 and 4) suggested that variables associated with relapse included: age, ethnicity, HCV RNA at baseline, cirrhosis, time to first HCV RNA undetectable, HCV RNA reduction at weeks 4, 12 and 24, and average HCV RNA reduction at nadir (≥0.5 log_10_ per month).

**Table 3:** Relationship of baseline predictors with relapse

Characteristic	Patients, *n*	Relapse, *n* (%)	OR (95% CI)*	*P*-value*
Sex				
Female†	181	57 (31.5)	1.00	
Male	358	120 (33.5)	1.10 (0.75–1.61)	0.636
Age, years				
18–30†	31	7 (22.6)	1.00	
>30–40	125	33 (26.4)	1.23 (0.48–3.12)	0.664
>40–50	215	67 (31.2)	1.55 (0.64–3.78)	0.334
>50–60	135	54 (40.0)	2.28 (0.92–5.67)	0.075
>60	33	16 (48.5)	3.22 (1.09–9.53)	0.034
Weight, kg				
<75†	204	67 (32.8)	1.00	
≥75	335	110 (32.8)	1.00 (0.69–1.45)	1.000
BMI‡, kg/m^2^				
≤27†	279	88 (31.5)	1.00	
27–30	119	44 (37.0)	1.27 (0.81–2.00)	0.292
>30	134	42 (31.3)	0.99 (0.64–1.54)	0.968
Race/ethnicity				
Non-Latino white†	380	117 (30.8)	1.00	
Latino white	112	46 (41.1)	1.57 (1.01–2.42)	0.043
African American	28	8 (28.6)	0.90 (0.38–2.10)	0.806
Other	19	6 (31.6)	1.04 (0.38–2.80)	0.942
ALT				
≤3 × ULN†	412	139 (33.7)	1.00	
>3 × ULN	127	38 (29.9)	0.84 (0.54–1.29)	0.424
HCV RNA, IU/mL				
≤400 000†	78	13 (16.7)	1.00	
>400 000–800 000	59	21 (35.6)	2.76 (1.24–6.14)	0.013
>800 000	402	143 (35.6)	2.76 (1.47–5.18)	0.002
Cirrhotic classification				
Noncirrhotic†	459	141 (30.7)	1.00	
Cirrhotic	80	36 (45.0)	1.85 (1.14–2.99)	0.013

ALT, alanine aminotransferase; BMI, body mass index; CI, confidence interval; HCV, hepatitis C virus; OR, odds ratio; ULN, upper limit of normal.

* From simple logistic regression that includes only one variable in the model.

† Reference.

‡ Missing data, *n* = 7.

**Table 4:** Relationship of hepatitis C virus (HCV) reduction variables with relapse

HCV reduction variable	Patients, *n*	Relapse, *n* (%)	OR (95% CI)*	*P*-value*
HCV RNA being undetectable for the first time				
In 5–12 weeks†	388	89 (22.9)	1.00	
In 13–24 weeks	151	88 (58.3)	4.69 (3.14–7.01)	<0.0001
HCV reduction at week 4 (log_10_)				
<1 log	51	34 (66.7)	6.37 (3.15–12.90)	<0.0001
≥1 log and <2 log	120	46 (38.3)	1.98 (1.15–3.40)	0.013
≥2 log and <3 log	144	47 (32.6)	1.54 (0.91–2.62)	0.107
≥3 log and <4 log†	134	32 (23.9)	1.00	
≥4 log and <5 log	70	13 (18.6)	0.73 (0.35–1.50)	0.386
HCV reduction at week 12 (log_10_)				
<1 log	5	3 (60.0)	2.19 (0.35–13.75)	0.403
≥2 log and <3 log	32	22 (68.8)	3.21 (1.36–7.56)	0.008
≥3 log and <4 log†	91	37 (40.7)	1.00	
≥4 log and <5 log	152	43 (28.3)	0.58 (0.33–1.00)	0.048
≥5 log and <6 log	183	56 (30.6)	0.64 (0.38–1.09)	0.099
≥6 log and <7 log	72	12 (16.7)	0.29 (0.14–0.62)	0.001
HCV reduction at week 24 (log_10_)				
≥3 log and <4 log†	35	6 (17.1)	1.00	
≥4 log and <5 log	153	49 (32.0)	2.28 (0.89–5.84)	0.087
≥5 log and <6 log	239	89 (37.2)	2.87 (1.15–7.18)	0.024
≥6 log and <7 log	102	30 (29.4)	2.01 (0.76–5.35)	0.160
Average HCV reduction at nadir (log_10_ per month)				
≥0.5 log and <1 log†	121	69 (57.0)	1.00	
≥1 log and <1.5 log	101	32 (31.7)	0.35 (0.20–0.61)	0.0002
≥1.5 log and <2 log	245	64 (26.1)	0.27 (0.17–0.42)	<0.0001
≥2 log and <2.5 log	72	12 (16.7)	0.15 (0.07–0.31)	<0.0001

CI, confidence interval; OR, odds ratio.

* From simple logistic regression that includes only one variable in the model.

† Reference.

Using a backward selection procedure starting with the full model, several less complex multivariable logistic models containing fewer variables were developed to predict relapse. The variables included in the optimal model and the parameter estimates are presented in Table 5.

**Table 5:** Parameter estimates of log odds of relapse for the variables included in the optimal model

Variable	Parameter estimate	Standard error	*P*-value
Intercept	−1.350	1.2435	0.278
Age: >50 *vs*≤50 years	0.451	0.237	0.057
Race: African American *vs* non-Latino white/other	4.473	2.205	0.043
Race: Latino white *vs* non-Latino white/other	−0.936	0.490	0.056
Time to undetectability: 24-week undetectable *vs* cEVR	−1.315	0.611	0.031
ALT quotient: per 1-unit increase	−0.319	0.115	0.006
ALT quotient × race: African American	−3.615	1.551	0.020
ALT quotient × race: Latino white	0.696	0.200	<0.001
ALT quotient × time to undetectability: 24-week undetectable	0.514	0.194	0.008
Baseline HCV (log_10_ IU/mL): per 1-unit increase	0.806	0.222	<0.001
HCV reduction rate: log_10_ baseline/log_10_ week 4: per 1-unit increase	−1.583	0.302	<0.001
HCV reduction rate: log_10_ week 4/log_10_ week 12: per 1-unit increase	−0.427	0.123	<0.001
Percentage peginterferon alfa-2a dose reduction*: per 1% reduction	0.028	0.011	0.010
Percentage ribavirin dose reduction*: per 1% reduction	0.020	0.008	0.008

ALT, alanine aminotransferase; cEVR, complete early virologic responders; HCV, hepatitis C virus.

* Because of safety reasons.

### Evaluation of the optimal model

**Fig 1:**
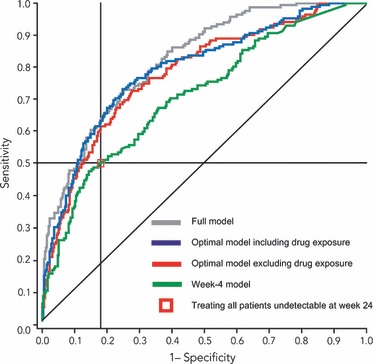
Receiver operating characteristic curve analysis of the selected models. See Table 1 for details of predictors included in the models.

A ROC curve derived from the optimal model was far from the diagonal straight line indicating that the optimal model was valuable in predicting relapse (Fig. 1). It is known that the sensitivity increases and the specificity decreases as a lower cut-off point is chosen for the predicted probability in the model (i.e. more patients are predicted as relapse). Therefore, it is necessary to select a reasonable cut-off point when making a prediction to balance between the gain in sensitivity and the loss in specificity. When using a cut-off point of 0.509 in predicted probability, the optimal model had a sensitivity of 50% and a specificity of 89%. When using a cut-off point of 0.406 in predicted probability, the optimal model had a sensitivity of 63% and a specificity of 82%.

### Performance of the optimal model compared with methods in practice

Current treatment guidelines do not identify a population that would benefit from intensified therapy nor do they recommend a preferred intensification strategy. Although several approaches to intensify therapy exist, the strategy of extending treatment duration is supported by several studies [10–12] and is easily implemented at a later stage in treatment. As practitioners adhere closely to straightforward guidelines, the optimal model was compared with potential methods in clinical practice. One possible method would be to intensify treatment for all patients that become HCV RNA undetectable between weeks 13 and 24 (slow responders) and assume that these patients have a high probability of relapse; patients with cEVR would not receive intensified treatment after week 48 because of their high probability of achieving SVR. A second and simpler method would be to intensify treatment for all patients with a <2 log reduction in HCV RNA at week 4 and allow all other patients to receive the standard 48-week treatment. When applying these two methods to the analysis sample, the first method was more accurate than the second method, providing a sensitivity of 50%*vs* 47%, respectively, and a specificity of 82%*vs* 74%, respectively (Table 6). Compared with the practice of intensifying treatment in all patients who became HCV RNA undetectable between weeks 13 and 24, the optimal model provided higher specificity (by 7%) when the sensitivities were the same and higher sensitivity (by 13%) when the specificities were the same. Thus, the optimal model predicted 7% more true SVR or 13% more true relapse than intensifying treatment for all slow responders.

**Table 6:** Comparison of selected models with treating all 24-week undetectable patients (slow responders) and treating all patients with week 4 hepatitis C virus (HCV) RNA <2 log reduction

			Same sensitivity*		
	
	Treating all slow responders	Treating all patients with week 4 HCV RNA <2 log reduction	Full model (*P*^†^ = 0.499)	Optimal model (*P*^†^ = 0.509)	Week 4 model (*P*^†^ = 0.384)
Sensitivity, %	50	47	50	50	50
Specificity, %	82	74	88	89	83
			Same specificity*		
			
			Full model (*P*^†^ = 0.395)	Optimal model (*P*^†^ = 0.406)	Week 4 model (*P*^†^ = 0.382)
			
Sensitivity, %	50	47	63	63	50
Specificity, %	82	74	82	82	82

* Fixed sensitivity or specificity as treating all patients who became undetectable at week 24 (clinical practice). ^†^Cut-off point for predicted probability. Patients with predicted probability greater or equal to the cut-off are predicted as relapse.

## Discussion

As approximately 50% of patients with HCV genotype 1 fail to achieve SVR after 48 weeks of peginterferon alfa-2a plus weight-based ribavirin treatment, strategies to improve these outcomes are imperative. Although some patients will fail to achieve EVR and will discontinue therapy, most patients will achieve this virologic milestone. However, a subset of EVR patients will experience relapse once treatment is completed. It is this subset of patients that are most difficult to identify prior to treatment completion and who will most likely benefit from an aid to improve their early identification so that more intensive treatment regimens may be used to improve SVR rates.

Using candidate predictors (baseline factors and viral kinetic variables) that are known to influence HCV therapeutic end-points, we developed a model that improved the accuracy of identifying non-RVR patients who responded by week 24 yet were unable to achieve SVR in this retrospective cohort. The model could identify a subset of patients at high risk for relapse and who could then be considered for intensified therapy. Thus, the use of this model could potentially lead to an increase in SVR rates as well as prevent unnecessary intervention in those patients who would not relapse even without intensified therapy.

Several strategies that intensify therapy have been explored to improve therapeutic outcomes. Extending treatment duration beyond 48 weeks is one such strategy that has been recently investigated. However, if applied to the majority of patients, this regimen will potentially place many patients at risk for unnecessary treatment-related adverse events. Not all investigators agree that extending treatment duration is an effective strategy for the majority of patients. Berg and colleagues [10] showed that there was no difference in SVR rates among groups of genotype 1-infected patients treated with peginterferon alfa-2a 180 μg/week plus ribavirin 800 mg/day for either 48 (*n* = 230) or 72 (*n* = 225) weeks. However, a subanalysis of this cohort revealed that those patients with detectable HCV RNA at week 12 experienced higher SVR rates with 72 weeks of therapy compared with those patients who received only 48 weeks of therapy (29%*vs* 17%; *P* = 0.04) [10]. A similar study by Sánchez-Tapias *et al.* [12] supports these findings. Patients with mixed HCV genotype (*n* = 326) and without RVR were randomized to receive either 48 or 72 weeks of peginterferon alfa-2a 180 μg/week plus ribavirin 800 mg/day. Although EOT responses were similar, non-RVR patients with HCV genotype 1 who received 72 weeks of therapy were much less likely to relapse than patients with 48 weeks of therapy (17%*vs* 53%; *P* = 0.002), resulting in a significant difference in SVR rates between the two groups (44%*vs* 28%; *P* = 0.003) [12]. Although the dose of ribavirin was suboptimal in both of these studies, the results suggest that a subset of HCV-infected patients benefit from extended treatment duration and that these patients can be identified while on treatment.

Accurately identifying candidates for intensified therapy is crucial to minimize both cost and morbidity. We developed a multiple logistic regression model that could identify non-RVR patients likely to relapse during the 24-week follow-up period after treatment is completed. The optimal model provided acceptable sensitivity and specificity to identify patients who may relapse and therefore may benefit from intensified therapy. Age, ethnicity, time to first HCV RNA undetectable, baseline ALT level, baseline HCV RNA level, advanced fibrosis and the degree of HCV RNA reduction at weeks 4 and 12 contributed to the risk for relapse and were included in the model. However, as computation is slightly laborious, the optimal model was compared with the more simplistic but clinically practical approach of intensifying therapy in all patients who are slow to respond to combination therapy and become HCV RNA undetectable between weeks 13 and 24. This approach has also been prospectively evaluated. Pearlman and colleagues [11] randomized treatment-naïve HCV genotype 1-infected patients with slow response (defined as achieving at least a 2-log decrease in HCV RNA from baseline, yet having detectable HCV RNA at 12 weeks and undetectable HCV RNA at 24 weeks) to 48 or 72 weeks of therapy with peginterferon alfa-2b 1.5 μg/kg/week plus ribavirin 800–1400 mg/day. While the EOT response rates were similar (48% for the 72-week arm *vs* 45% for the 48-week arm; *P*-value not significant), the rate of SVR was superior in patients treated for 72 weeks (38%*vs* 18%; *P* = 0.026) [11]. More recent studies [15–17], including a meta-analysis of six randomized controlled trials [18], support the observation that extended treatment duration may improve therapeutic efficacy in certain subgroups of patients with a slow response to HCV therapies. However, it is important to note the differences in study designs when comparing these studies because others have shown conflicting results [19]. In our analysis, the practice of treating all patients without cEVR but undetectable HCV RNA between weeks 13 and 24 was less accurate in predicting relapse when compared with the optimal model, but may be the most practical and straightforward to implement clinically.

Recent studies have identified genetic variations in or near the interleukin 28B (*IL28B*) gene that strongly predict the response to current HCV therapy [20–22]. The utility of *IL28B* genotyping in identifying patients likely to respond to HCV therapy is expected to improve patient care in the near future. Multiple logistic analyses that included baseline (pretreatment) clinical predictors of viral response showed that several *IL28B* genetic polymorphisms were independent predictors of response [20–22]. Subsequent studies have confirmed that the *IL28B* rs12979860 CC genotype was the strongest pretreatment factor associated with SVR and had a higher adjusted odds ratio than HCV genotype, viral load, age, sex, race, fibrosis or previous treatment status [23,24]. This genotype also predicted SVR with 78% specificity and 65% sensitivity in patients infected with HCV genotype 1 [23]. These studies indicate that the predictive value of *IL-28B* for SVR is limited to its use as a baseline pretreatment characteristic only. It is well known that the on-treatment virologic response to HCV therapy is a stronger predictor for SVR than baseline characteristics [5]. For example, approximately 90% of patients with RVR achieve a SVR, making RVR the strongest predictor of virologic response [5,6,9]. In addition, it is suggested that the rs12979860 CC genotype is associated with improved viral kinetics [24]. One study showed that this genotype was associated with lower rates of relapse [24]; however, this genotype did not accurately distinguish between patients with relapse and patients with SVR [23]. Thompson *et al.* also reported that although this genotype did not contribute additional information to patients with RVR, the CC genotype added significant predictive value in patients with non-RVR; however, once the week-12 virologic response was determined, the predictive utility of this genotype was very weak [24]. Thus, additional studies are required to confirm the independent predictive value of this marker. It would have been of interest to evaluate the effect of the *IL28B* genotype on the proposed models in our study; however, because DNA samples were not collected during the trials included in this analysis, we cannot comment on what the effect of adding *IL28B* polymorphisms to our models would have had on their specificity or predictability.

There are advantages to the early identification of a patient in need of alternative therapeutic strategies. Combination therapy is associated with significant adverse events that affect family planning, employment and health-related quality of life. Although RVR is highly correlated with SVR, early viral kinetics have not been used to predict suboptimal response. The week-4 model of intensifying treatment in all patients with a <2 log decrease in HCV RNA at week 4 and who were HCV RNA undetectable by week 24 was the earliest marker of candidates for intensified therapy, but was also the least accurate. However, this time point may be advantageous if considering alternative therapeutic strategies such as high-dose ribavirin or the addition of a third agent. Although the proposed model was developed using data from patients treated with peginterferon alfa-2a plus ribavirin, given the fact that 20–30% of patients relapse after treatment with either of the currently approved pegylated interferons, it is likely that this model is applicable to all patients with HCV genotype 1 undergoing standard HCV therapy [25].

In conclusion, two steps are necessary to achieve the goal of increasing the therapeutic efficacy in patients who may relapse on standard HCV therapy. First, the patient would have to be accurately differentiated from those patients who are likely to achieve SVR on standard therapy alone. Second, the identified patient would then have to receive an intensified therapy that has been proven in prospective clinical trials to increase therapeutic efficacy. The multiple logistic regression model developed in this study accomplishes the first step of identifying non-RVR patients who may relapse during the 24-week follow-up period after treatment with standard of care of combination peginterferon alfa plus ribavirin. However, the external validity of this model can only be achieved by evaluating the sensitivity and specificity after applying it to a separate database of patients who received similar treatments. This process will ensure the robustness of the model and that this model can be generalized to include other interferon-based therapies. The model predicts those patients at risk for suboptimal response at a time when the on-treatment regimen may still be modified by strategies to intensify therapy and improve treatment outcomes. Once the applicability of the model across all interferon-based therapies is established, these results may guide individualization of treatment decisions in clinical practice, and ultimately, the model may be used to develop a nomogram that will assist physicians in determining whether to treat a patient with intensified therapy.

## Statement of interests

Authors’ declaration of personal interests: Nancy Reau has received research support from Human Genome Sciences. Fayez M. Hamzeh is an employee of Genentech. Ellen Lentz is an employee of Genentech. Xiaolei Zhou has received funding/contracts from Roche and Genentech as an employee of RTI Health Solutions. Donald M. Jensen has served as a consultant and an advisory board member for Roche, Vertex, Abbott, Human Genome Sciences, GlobeImmune and Boehringer-Ingelheim, and has received research funding from Roche, Vertex, Tibotec and Boehringer-Ingelheim. Declaration of funding interests: This study was funded in full by Roche. The writing of this paper was funded in part by Genentech, Inc. Initial data analyses were undertaken by Jianmin Wang, PhD, who is an employee of RTI Health Solutions for statistical consultation, funded by Roche. Writing support was provided by Paul MacCallum, PhD of Envision Scientific Solutions, Ltd. and funded by Genentech, Inc.

## Abbreviations

ALT: alanine aminotransferase
cEVR: complete early virologic response
CHC: chronic hepatitis C
EOT: end of treatment
EVR: early virologic response
HCV: hepatitis C virus
ROC: receiver operating characteristic
RVR: rapid virologic response
SVR: sustained virologic response
